# A Novel Gene Signature Based on CDC20 and FCN3 for Prediction of Prognosis and Immune Features in Patients with Hepatocellular Carcinoma

**DOI:** 10.1155/2022/9117205

**Published:** 2022-03-30

**Authors:** Xing Lai, Ya-kun Wu, Guo-qing Hong, Jun-ke Li, Qing Luo, Jin Yuan, Guo-hua Dai, Shuang-quan Liu, Hua-guo Feng

**Affiliations:** ^1^Department of Hepatobiliary Surgery, The Tongnan District People's Hospital, Chongqing, China; ^2^Department of Hepatobiliary Surgery, The Suining Central Hospital, Suining, China; ^3^Department of Infection Diseases, the Chongqing University Jiangjin Hospital, School of Medicine, Chongqing University, Chongqing, China; ^4^Department of Hepatobiliary Surgery, the Chongqing University Jiangjin Hospital, School of Medicine, Chongqing University, Chongqing, China

## Abstract

Long-term survivals of patients with hepatocellular carcinoma (HCC) remain unfavorable, which is largely attributed to active carcinogenesis. Growing studies have suggested that the reliable gene signature could act as an independent prognosis factor for HCC patients. We tried to screen the survival-related genes and develop a prognostic prediction model for HCC patients based on the expression profiles of the critical survival-related genes. In this study, we analyzed TCGA datasets and identified 280 genes with differential expressions (125 increased genes and 155 reduced genes). We analyzed the prognosis value of the top 10 dysregulated genes in HCC patients and identified three critical genes, including FCN3, CDC20, and E2F1, which were confirmed to be associated with long-term survival in both TCGA and ICGC datasets. The results of the LASSO model screened CDC20 and FCN3 for the development of the prognostic model. The CDC20 expression was distinctly increased in HCC specimens, while the FCN3 expression was distinctly decreased in HCC. At a suitable cutoff, patients were divided into low-risk and high-risk groups. Survival assays revealed that patients in high-risk groups exhibited a shorter overall survival than those in low-risk groups. Finally, we examine the relationships between risk score and immune infiltration abundance in HCC and observed that risk score was positively correlated with infiltration degree of B cells, T cell CD4+ cells, neutrophil, macrophage, and myeloid dendritic cells. Overall, we identified three critical survival-related genes and used CDC20 and FCN3 to develop a novel model for predicting outcomes and immune landscapes for patients with HCC. The above three genes also have a high potential for targeted cancer therapy of patients with HCC.

## 1. Introduction

Hepatocellular carcinoma (abbreviated as HCC) refers to the third most significant cause of deaths associated with cancer in the world [1]. The primary risk factors driving hepatocellular carcinoma have included hepatitis B and C viral infections, alcoholic liver disease, and nonalcoholic fatty liver disease [2, 3]. Patients suffering from the advanced stage of HCC, receiving the molecularly targeted drug sorafenib, were found with a greater median survival as compared with those administrated with the placebo, as demonstrated by existing research [4, 5]. However, the targeted therapy of HCC still has numerous limitations. For the improvement of HCC patients' prognosis, it is particularly necessary to develop and identify the novel key biomarkers for treatments of the patients with HCC.

Numerous genetic alternations influence HCC progression and indicate HCC prognosis [6, 7]. Many cellular genetic and molecular aberrations have been identified, which form the basis of classification and risk stratification for HCC [8, 9]. Due to differences in treatment regimens and survival, it is important for existing studies to identify molecular biomarkers that are critical for the development and progression of HCC [10, 11]. In addition, the identification of biomarkers and subsequent development of targeted therapies may improve clinical outcomes. Over the past few years, RNA sequencing has become a powerful method for screening transcripts [12, 13]. The gene expression profile involved in HCC has been extensively studied, providing useful ideas of the molecular mechanism of HCC [14, 15]. The present study is aimed at exploring the possible prognosis biomarkers for HCC patients via analyzing TCGA and ICGC datasets.

## 2. Materials and Methods

### 2.1. Evidence from TCGA and ICGC Database

In this study, the TCGA database was utilized in terms of HCC for the acquisition of information relating to relevant clinical data (data types: clinical supplement), gene expressions (workflow type: HTSeq-FPKM), and immune system infiltrates. The present study followed TCGA's publication guidelines. In addition, we collected the microarray data of 240 HCC samples and 202 nontumor samples from ICGC datasets.

### 2.2. Identification of mRNA with Differential Expressions in HCC

Based on transcripts per million approaches, the data of raw count were first normalized and then received a log2 transformation. Subsequently, we annotated 19654 protein-coding genes. With the use of the limma version 3.36.2 *R* package, the genes with differential expressions (DEGs) were determined. DEGs with an adjusted *p* value of<0.05 and an absolute log2 fold change (FC) > 4 were screened to perform the following investigation.

### 2.3. Survival Investigation

Kaplan-Meier survivals were applied to examine the survival differences between the low-/high-risk groups used the above datasets. The ‘survival' package in R (http://cran.r-project.org/package=survival) was applied to carry out a log-rank test and univariate assays.

### 2.4. Establishment of the Prognosis Gene Signature

We collected the sequencing data and clinical data from TCGA datasets. All patients with a follow-up period <60 days were excluded for survival tests. LASSO methods were carried out to construct a prognosis signature. Cox regression model coefficients (*M*) multiplied with the levels of genes: the risk scores = (*M* gene 1 × levels of gene 1) + (*M* gene 2 × levels of gene 2) + (*M* gene 3 × levels of gene 3) + ⋯+(*M* gene *n* × levels of gene *n*). The optimal cut-off values were determined by the use of survminer *R* package. Finally, Kaplan-Meier tests were carried out to study the prognosis value of risk score in HCC patients.

### 2.5. Functional Enrichment Investigation

Gene Ontology (GO) and Kyoto Encyclopedia of Genes and Genomes (KEGG) pathway enrichment investigation were performed between HCC specimens and nontumor specimens by the use of the “clusterProfiler” *R* package [16]. GO terms and KEGG pathways with *p* values <0.05 had statistical significance.

### 2.6. Tumor IMmune Estimation Resource (TIMER) Database Investigation

We analyzed risk score and the correlation of risk score with the abundance of infiltrating immune cells in HCC patients via the TIMER algorithm databases [17]. As an important element, tumor purity influenced the investigations of immune infiltration in HCC specimens via genomic approaches.

### 2.7. Statistical Investigation

Statistical assays were carried out by the use of *R* (version 4.0.2, RStudio Inc., Boston, MA, USA) software packages. Student's *t*-test was carried out to compare whether genes exhibited a dysregulated level between HCC specimens and nontumor specimens. Log-rank tests were carried out to compare the possible differences between low and high-risk groups. Kaplan-Meier curve was carried out for the visualization the survivals. Receiver operating characteristic (ROC) tests were carried out for the determination of the accuracy of the prognosis signature applying the *R* “timeROC” package. The value of *p* < 0.05 and FDR < 0.05 had statistical significance.

## 3. Results

### 3.1. Identification of Genes with Differential Expressions in HCC

**Figure 1:**
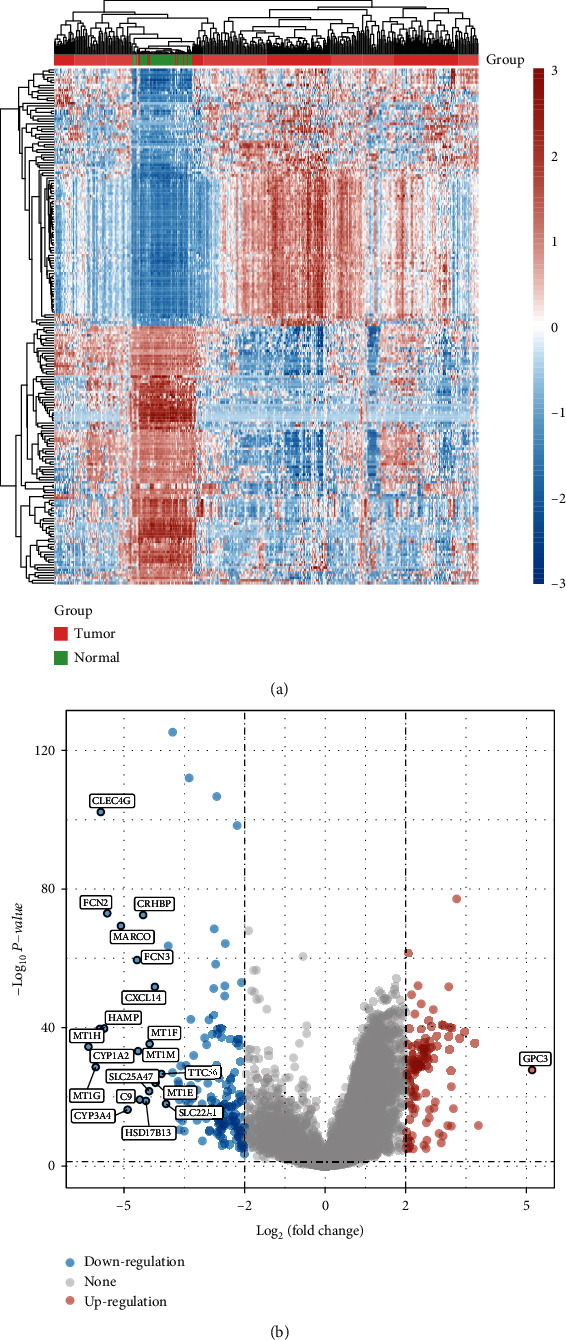
DEGs identified from TCGA datasets. (a) Heatmap of the all DEGs. (b) Volcano map of DEGs between HCC specimens and nontumor specimens.

**Figure 2:**
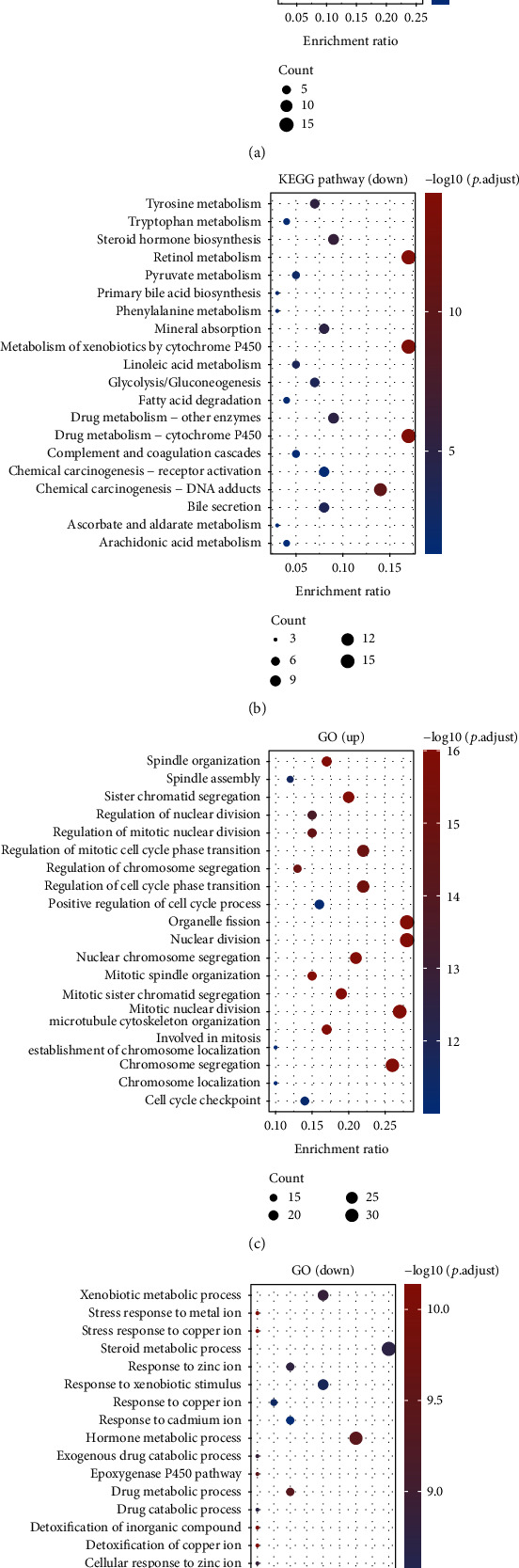
Functional investigation in accordance with the DEGs between normal and tumor groups in the TCGA group. (a) Barplot graph in terms of KEGG pathways. (b) Bubble graph in terms of GO enrichment using 125 increased genes. (c) Barplot graph in terms of KEGG pathways. (d) Bubble graph in terms of GO enrichment using 155 reduced genes.

To analyze genes with differential expressions in HCC, we analyzed TCGA datasets and identified 280 genes with differential expressions (125 increased genes and 155 reduced genes) in HCC using log2 (FC) > 4 and *p* < 0.05. Heat map (Figure 1(a)(and Volcanic map (Figure 1(b)) showed the expressing pattern of the genes with differential expressions in HCC. Then, we performed GO and KEGG tests using the above 280 genes. As shown Figure 2(a), KEGG tests revealed that 125 increased genes were mainly enriched in p53 signaling pathway, viral carcinogenesis, pyrimidine metabolism, and microRNAs in cancer. 155 reduced genes were mainly enriched in tyrosine metabolism, tryptophan metabolism, steroid hormone biosynthesis, and retinol metabolism (Figure 2(b)). GO tests revealed that the 125 increased genes were mainly involved in spindle organization, spindle assembly, sister chromatid segregation, and regulation of nuclear division (Figure 2(c)), and 155 reduced genes were mainly involved in xenobiotic metabolic process, stress response to metal ion, stress response to copper ion, and steroid metabolic process (Figure 2(d)).

### 3.2. The Prognosis Value of the Top 10 Dysregulated Genes in HCC Patients

**Figure 3:**
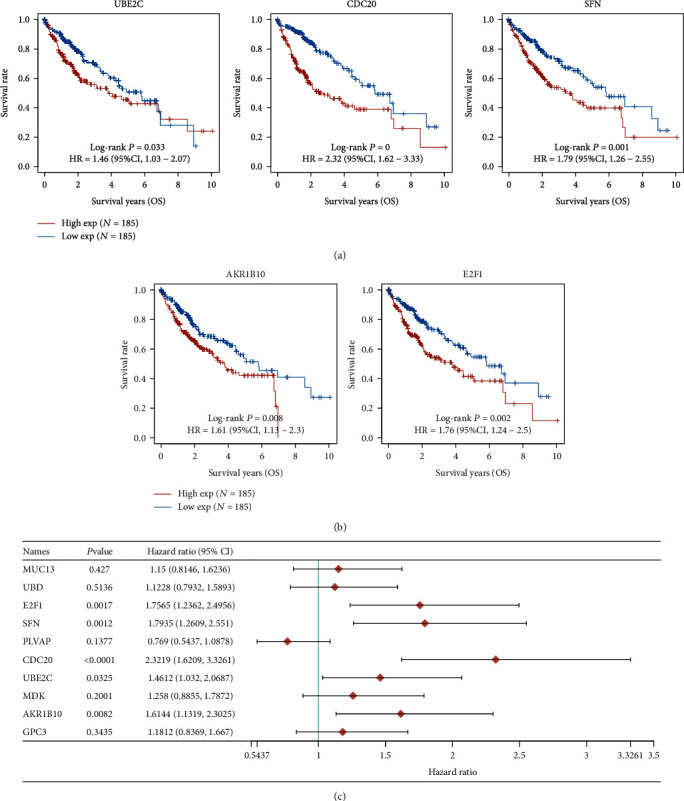
Identification of total genes having a correlation with survival in HCC using the top 10 overexpressed genes. (a, b) UBE2C, CDC20, SFN, AKR1B10, and E2F1 were identified to be high risk factors via Kaplan-Meier tests. (c) Univariate Cox regression analyses.

**Figure 4:**
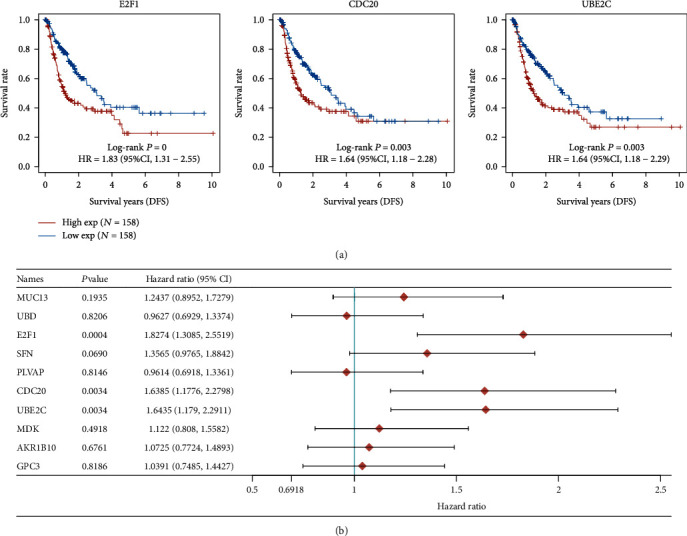
Identification of disease-free genes having a correlation with survival in HCC using the top 10 overexpressed genes. (a) E2F1, CDC20, and UBE2C were identified to be high risk factors via Kaplan-Meier tests. (b) Univariate Cox regression analyses.

**Figure 5:**
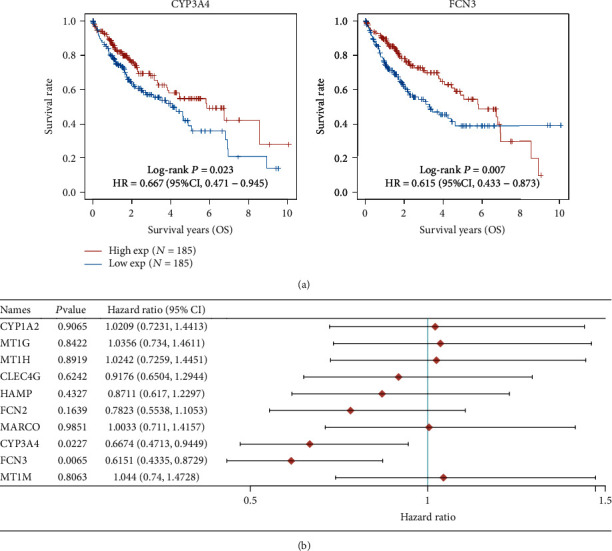
Identification of total genes having a correlation with survival in HCC using the top 10 reduced genes.(a) Patients with high expression of CYP3A4 and FCN3 showed a longer total survival as compared with those having low expressions of CYP3A4 and FCN3. (b) Univariate Cox regression analyses.

**Figure 6:**
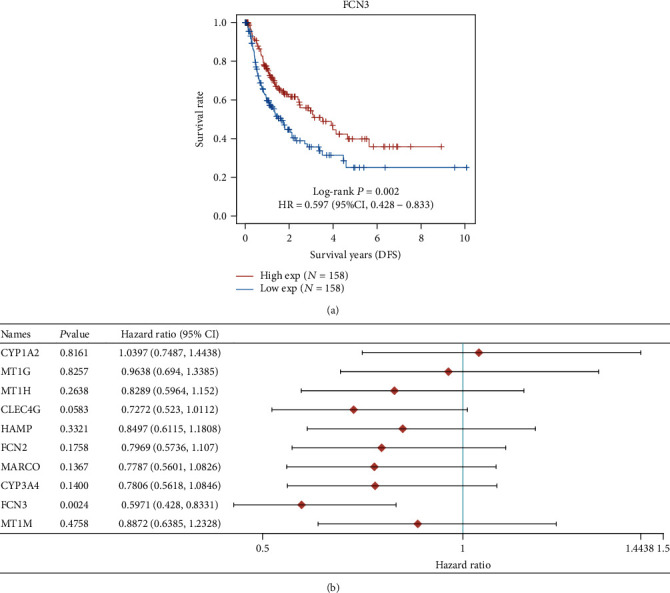
Identification of disease-free genes having a correlation with survival in HCC using the top 10 reduced genes. (a) Low expression of FCN3 had a correlation with poor prognosis of HCC patients. (b) Univariate Cox regression analyses.

**Figure 7:**
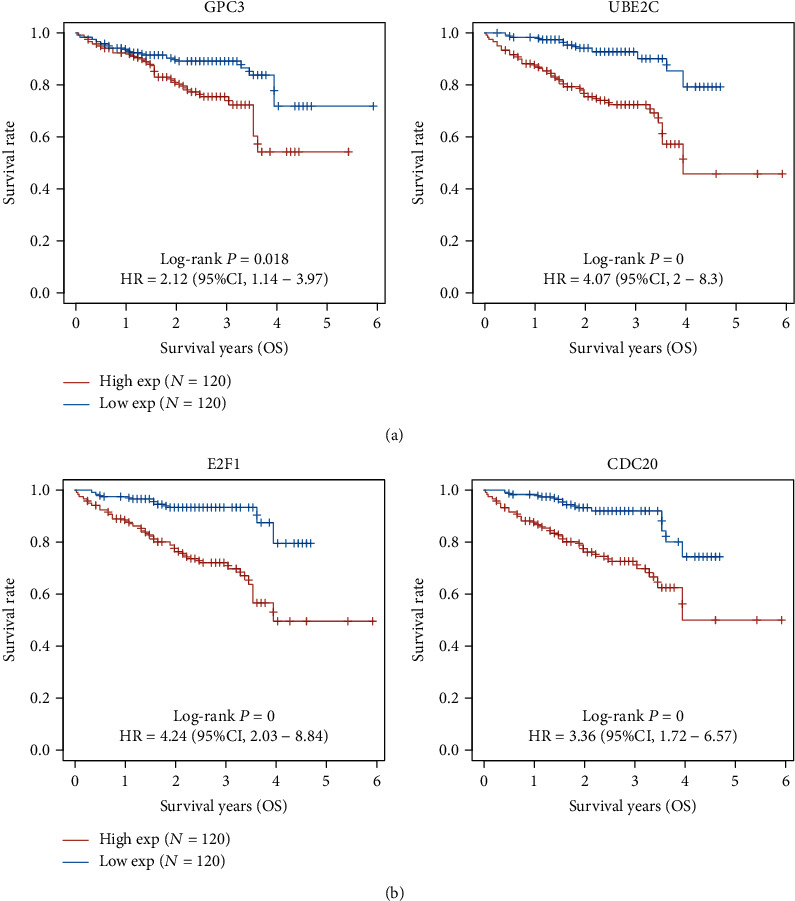
The prognosis value of the top 10 overexpressed genes in HCC from ICGC datasets. (a, b) CPC3, UBE2C, E2F1, and CDC20 were identified as OS-related genes.

**Figure 8:**
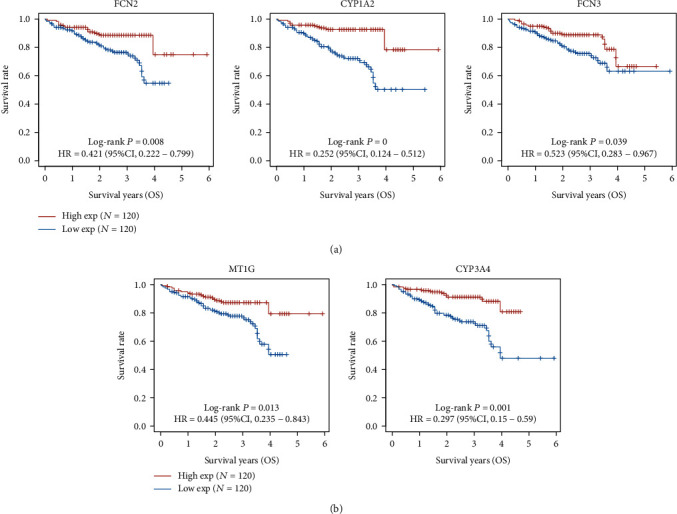
The prognosis value of the top 10 reduced genes in HCC from ICGC datasets. (a, b) FCN2, CYP1A2, FCN3, MT1G, and CYP3A4 were identified as OS-related genes.

To screen possible prognosis biomarkers in HCC patients, we analyzed the association between the top 10 dysregulated gene and total survivals of HCC patients using TCGA datasets. All HCC patients were divided into two groups (high and low) based on the mean expression of FCN3, CDC20, and E2F1. We observed that the high expression of UBE2C, CDC20, SFN, AKR1B10, and E2F1 had a correlation with a shorter total survival (Figures 3(a) and 3(b)), which was also confirmed by the use of univariate Cox regression analyses (Figure 3(c)). In addition, we found that the high expression of E2FF1, CDC20, and UBE2C had a correlation with a shorter disease-free survival in HCC patients (Figure 4(a)), which was also confirmed by the use of univariate Cox regression analyses (Figure 4(b)). Then, we analyzed the top 10 highly expressed genes in HCC and observed that the high expression of CYP3A4 and FCN3 in HCC specimens predicted a longer total survival (Figure 5(a)), which was also confirmed by the use of univariate Cox regression analyses (Figure 5(b)). Moreover, we found that just the FCN3 expression had a correlation with disease-free survival of HCC patients, which was also confirmed by the use of univariate Cox regression analyses (Figures 6(a) and 6(b)). In order to further demonstrate their prognosis value in HCC, we further downloaded ICGC datasets, including 240 HCC patients. The results of survival tests revealed that the high expression of GPC3, UBE2C, E2F1, and CDC20 had a correlation with a shorter total survival in HCC patients (Figures 7(a) and 7(b)). In addition, the high expression of FCN2, CYP1A2, FCN3, MT1G, and CYP3A4 had a correlation with a longer total survival in HCC patients (Figures 8(a) and 8(b)).

### 3.3. The Expression of the Critical Genes in HCC

**Figure 9:**
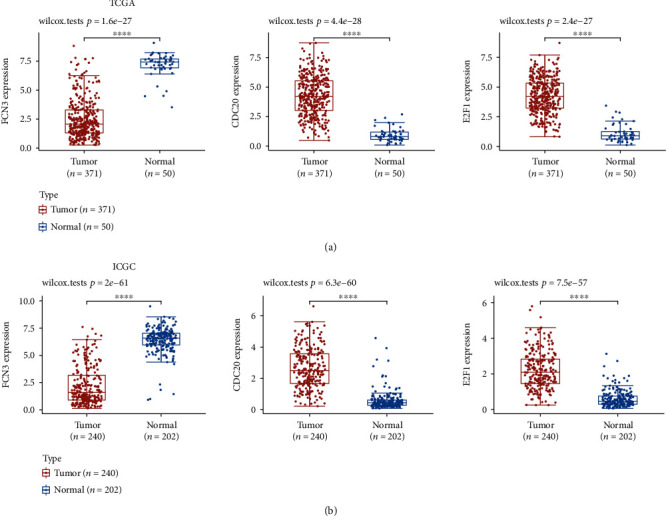
The expression of the critical genes having a correlation with survival in HCC. The FCN3 expression was noticeably reduced in HCC specimens, while CDC20 and E2F1 showed a decreased expression based on (a) TCGA datasets and (b) ICGC datasets.

We analyzed the prognosis value of the top 10 dysregulated genes in HCC patients and identified three critical genes, including FCN3, CDC20, and E2F1, which were confirmed to be associated with long-term survival in both TCGA and ICGC datasets. As shown in Figure 9(a), we found that the FCN3 expression was reduced in HCC specimens, while the expression of CDC20 and E2F1 was upregulated in HCC specimens. The above findings were further demonstrated by the use of ICGC datasets (Figure 9(b)).

### 3.4. Establishment of the Two-Gene-Based Prognosis Gene Signature in HCC Patients

**Figure 10:**
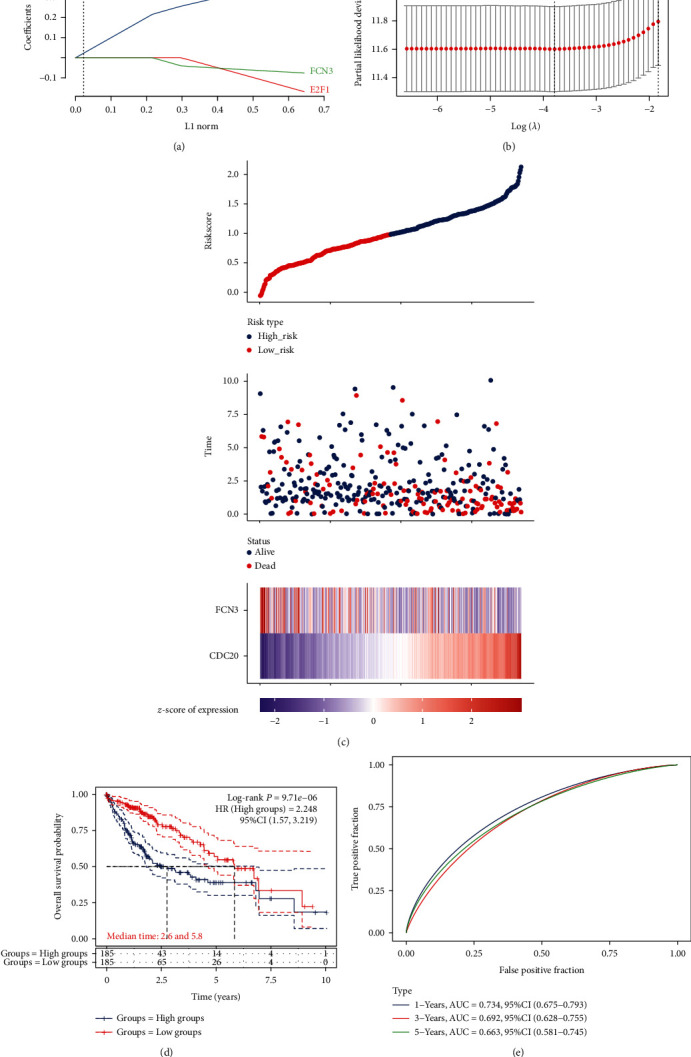
The construction of two gene signature and the assessment of its prognosis value. (a) LASSO coefficient profiles of the three genes having a correlation with survival. (b) We drew a coefficient profile plot against the log (lambda) sequence in the LASSO model. (c) The distributions of the status, survival time, and risk score of patients from TCGA datasets. (d) Kaplan-Meier curves of the gene signature from TCGA datasets. (e) The prognosis gene signature's time-dependent ROC curves from TCGA datasets.

The LASSO model was applied to screen the most useful prognosis genes from the above three genes, including FCN3, CDC20, and E2F1. The optimal gene signature consisting of two prognosis DEGs (i.e., CDC20 and FCN3) as well as the corresponding coefficients were identified (Figures 10(a) and 10(b)). Among three signature genes, CDC20 was promotive, and FCN3 was protective. The expressions of CDC20 and FCN3 and corresponding coefficients from the LASSO model were applied to examine the individual-level risk scores for all samples as following: risk score = −0.031 × expression of FCN3 + 0.2477 × expression of CDC20. After collecting two-gene-based risk scores, we further develop a prognosis classifier to divide HCC patients into two groups (high and low risk). The risk curve is plotted in Figure 10(c). Clinical tests revealed that patients in high-risk groups exhibited a shorter OS than those in low-risk groups (Figure 10(d)). The diagnostic value was shown in Figure 10(e).

### 3.5. Pertinence of Risk Score and Immune Infiltration Level in HCC

**Figure 11:**
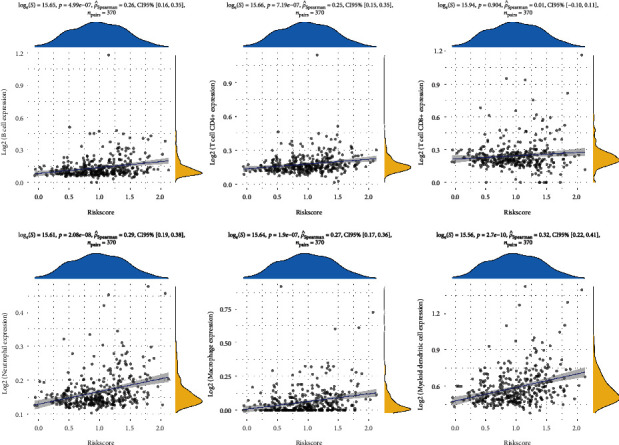
Relationship of risk score to immune infiltration level in HCC. Risk score has a distinct positive association to infiltrating levels of B cells, T cell CD4+ cells, neutrophil, macrophage, and myeloid dendritic cells in HCC.

The distribution of tumor-infiltrating lymphocytes has been demonstrated to be a critical predictor for tumor metastasis and long-term survivals of HCC patients [18, 19]. We carried out TIMER databases to examine the relationship between risk score and immune infiltration abundance in HCC and observed that risk score was positively correlated with infiltration degree of B cells, T cell CD4+ cells, neutrophil, macrophage, and myeloid dendritic cells (Figure 11).

## 4. Discussion

Cancer remains the major public health burden which counts for one in four deaths in the United States [20, 21]. The etiology of HCC is associated with complex risk factors including genetic factors, environmental factors, and virus infections, which are responsible for lacking of sensitive and robust biomarkers for the early detection of HCC [22, 23]. It is of great interest in identifying reliable and informative prognosis biomarkers for cancer patients to provide valuable information for clinical decision-making.

Thanks to TCGA and ICGC datasets, we had the ability to perform comprehensive investigation on many sequencing data of a large number of tumor samples. The present study performed *R* and identified 1991 increased genes and 573 reduced genes. KEGG tests revealed that these dysregulated genes were widely associated with tumor-related pathway, such as p53 signaling pathway, human immunodeficiency virus 1 infection, and glycolysis [24, 25]. Then, we focused on the top 20 abnormally expressed genes in HCC. Via a series of survival tests, we identified three critical genes, including CDC20, E2F1, and FCN3. The above three genes not only exhibited a dysregulated level in HCC but also had a correlation with total survival and disease-free survival in both TCGA and ICGC datasets. Our findings suggested them as novel prognosis biomarkers for HCC patients.

Previously, several studies have reported the function of the three genes in several tumors, including HCC. For instance, CDC20 was reported to be highly increased and served as an unfavorable prognosis marker in HCC samples. Knockdown of CDC20 noticeably promoted the radiation efficacies on the growth retardation in HCC cells via regulating Bcl-2/Bax signal, and the expressions of CDC20 were noticeably reduced due to the overexpression of P53 through radiation [26]. Yu et al. reported that E2F1, which was highly expressed in HCC, mediated DDX11 transcriptional activation facilitates HCC cells' invasion, migration, and proliferation via PI3K/AKT/mTOR pathway [27]. In addition, the potential of FCN3 acting as novel prognosis biomarker was also reported in several tumors, including HCC [28–30]. These findings indicated E2F1 and CDC20 as tumor promoters in HCC, which was consistent with our findings that they predicted a poor prognosis of HCC patients. On the contrary, we observed that FCN3 expression was noticeably reduced in HCC in both TCGA and ICGC datasets, and its downregulation predicted a poor prognosis. Our findings suggested the above genes many exhibited a different function on the progression HCC. Further experiments were needed to further demonstrate our findings.

Over the past few years, increasing studies found that the prognosis model based on several critical genes displayed a high sensitivity than a single gene. Several bioinformatic investigations in HCC have previously been carried out in accordance with wide perspectives. A four-gene signature (CENPA, SPP1, MAGEB6, and HOXD9) and a six-gene signature (GLS, SRXN1, SMG5, VNN2, AHSA1, and SQSTM1) were demonstrated to show an important value in predicting the total survivals of HCC patients [31, 32]. The present study performed LASSO regression model and identified CDC20 and FCN3 for the factors of developing the prognosis model. We observed that patients with high risk showed a shorter total survival as compared with those having a low risk. Importantly, according to the results of ROC tests, we found that the area under the curve (AUC) was 0.734 for one-year survival and 0.663 for three-year survival in the training group of TCGA, highlighting its potential used as a novel prognosis model for HCC patients.

Ordinarily, the immune system possesses a strong ability in eliminating tumor cells in tumor microenvironment [33]. Unfortunately, tumor cells have developed many powerful methods to avoid the attack of immune system. Tumor immunotherapy can enhance the ability of immune system targeting tumor cells, such as cellular treatments, therapeutic antibodies, and cancer vaccines [34, 35]. Tumor-infiltrating immune cells (TIICs) exhibited a functional effect on the clinical outcomes of patients in many types of tumors [36, 37]. In order to explore the associations between risk score and TIICs, TIMER was carried out. Importantly, we observed a distinct association between risk score and several types of immune cells, including B cells, T cell CD4+ cells, neutrophil, macrophage, and myeloid dendritic cells. Overall, our signature may be involved in immune responses via modulating immune cells.

Nevertheless, several limitations here are worth mentioning. First of all, LASSO may ignore some important factors affecting the prognosis of HCC in the process of adjusting the weight of regression coefficients. Secondly, the samples lacked some clinical follow-up information; so, this study did not consider the presence of other health conditions to distinguish prognostic biomarkers. Based on this, we need further genetic and experimental studies, larger samples, and experimental validation.

## 5. Conclusion

We identified three critical genes (FCN3, CDC20, and E2F1) involved tumor prognosis in HCC patients. They were demonstrated to exhibit a dysregulated level in HCC and may be used a new biomarker. We further developed a novel prognosis mode based on CDC20 and FCN3. The signature provides a novel insight into immune-related genes in HCC and identifies potential biomarkers for prognosis and immunotherapy.

## Data Availability

The data used to support the findings of this study are available from the corresponding authors upon request.
